# RESIDENT EXPERIENCES OF PERSON-CENTRED CARE AT MEALTIMES IN LONG-TERM RESIDENTIAL CARE: A RAPID ETHNOGRAPHY

**DOI:** 10.1093/geroni/igad104.0967

**Published:** 2023-12-21

**Authors:** Megan Davies, Franziska Zuniga, Hilde Verbeek, Sandra Staudacher

**Affiliations:** Medical Faculty, University of Basel, Basel, Basel-Stadt, Switzerland; University of Basel, Basel, Basel-Stadt, Switzerland; Maastricht University, Maastricht, Limburg, Netherlands; Universität Basel, Basel, Basel-Stadt, Switzerland

## Abstract

Poor nutrition is a common ongoing problem in long-term residential care, often resulting in reduced quality of life. Previous research has concluded that the content of the meal, dining environment, service style and general atmosphere all add to the mealtime experience, suggesting that person-centred mealtimes are optimal. However, knowledge about which elements of person-centred care can be achieved in a mealtime setting in a given context is currently lacking. As part of the TRANS-SENIOR research network, rapid ethnographies were conducted across multiple sites (including interviews, observations and informal conversations), in long-term residential care homes in the UK, Switzerland and the Netherlands between October 2020 and December 2021. During analysis and interpretation of data, different themes were developed where either successfully achieved or missed opportunities for person-centred moments were observed. We observed differences between the long-term care homes in as much as the setting could be considered, resident choice was implemented, residents were enabled, care was individualized in the communal setting and how much the person of the residents was known with their past and present. In the presentation we will discuss the possibility to provide person-centred care during mealtimes in view of differences between staff approaches, the overall environment (size of dining area, seating arrangements etc.), allocation of staff resources and country-specific rules and regulations. We see an interplay of factors in place for mealtimes to be moments of participatory choice, interaction, independence, and dignity.

